# The Role of NOX4 and TRX2 in Angiogenesis and Their Potential Cross-Talk

**DOI:** 10.3390/antiox6020042

**Published:** 2017-06-08

**Authors:** Chaofei Chen, Li Li, Huanjiao Jenny Zhou, Wang Min

**Affiliations:** 1 Center for Translational Medicine, The First Affiliated Hospital, Sun Yat-Sen University, Guangzhou 510080, China; chen_cf547@126.com (C.C.); lilihaha900107@163.com (L.L.); 2 Department of Pathology and the Vascular Biology and Therapeutics Program, Yale University School of Medicine, New Haven, CT 06520, USA; huanjiao.zhou@yale.edu

**Keywords:** angiogenesis, NOX4, TRX2, ROS

## Abstract

The nicotinamide adenine dinucleotide phosphate (NADPH) oxidase (NOX) family is the major source of reactive oxygen species (ROS) in the vascular system. In this family, NOX4, a constitutive active form of NOXs, plays an important role in angiogenesis. Thioredoxin 2 (TRX2) is a key mitochondrial redox protein that maintains normal protein function and also provides electrons to peroxiredoxin 3 (PRX3) to scavenge H_2_O_2_ in mitochondria. Angiogenesis, a process of new blood vessel formation, is involved in a variety of physiological processes and pathological conditions. It seems to be paradoxical for ROS-producing NOX4 and ROS-scavenging TRX2 to have a similar role in promoting angiogenesis. In this review, we will focus on data supporting the role of NOX4 and TRX2 in angiogenesis and their cross-talks and discuss how ROS can positively or negatively regulate angiogenesis, depending on their species, levels and locations. NOX4 and TRX2-mediated ROS signaling could be promising targets for the treatment of angiogenesis-related diseases.

## 1. Introduction

Angiogenesis, a process of new blood vessel formation, is involved in a variety of physiological processes and pathological conditions [1,2,3]. Excessive angiogenesis can cause cancer, diabetic retinopathy and atherosclerosis, while insufficient angiogenesis links peripheral arterial disease and myocardial infarction. It is reported that reactive oxygen species (ROS) can regulate angiogenesis in both positive and negative manners. In vascular cells, ROS are generated from a number of sources, including the nicotinamide adenine dinucleotide phosphate (NADPH) oxidases, xanthine oxidase, the uncoupling of NO synthase and mitochondria [2,3,4,5]. NADPH oxidases have been considered as the major sources of ROS in the vasculature [6]. Recent reports suggest that ROS generated from mitochondria gravely regulate endothelial cell (EC) function [2,3,7,8]. Numerous cardiovascular risk factors contribute to mitochondria malfunction, inducing overproduction of ROS. Under physiological conditions, ROS are known to serve as second messengers in signal transduction that regulate EC growth, proliferation, apoptosis, barrier function, vasodilatation and vascular remodeling [9,10,11]. This is well demonstrated from in vitro hypoxia and in vivo ischemia on angiogenesis. However, excessive ROS production resulting from mitochondrial dysfunction can inhibit reparative angiogenesis by inducing endothelial dysfunction and cell apoptosis under pathological conditions such as diabetes and myocardial infarction.

Angiogenesis is delicately co-regulated by ROS producing oxidases and ROS scavenging enzymes. NOX4 is the major isoform of NADPH oxidases expressed in vascular cells and predominantly produces ROS, which plays an important role in angiogenesis. Thioredoxin 2 (TRX2) is the main ROS-scavenging enzyme in mitochondria that balances the ROS levels and maintains mitochondrial function in various cells. TRX2 also positively regulates ischemia-induced angiogenesis. The aim of this review is to briefly summarize recent progress and information on the redox signaling in angiogenesis with a focus on NOX4 and TRX2.

## 2. NADPH Oxidase Family

The NADPH oxidase (NOX) family consists of seven members, including NOX1–5 and the dual oxidases (Duox) 1 and 2. NOX1, NOX2, NOX4 and NOX5 are expressed in the vascular system [12]. Except for uncoupled endothelial nitric oxide synthase (eNOS) and mitochondria, the major vascular sources of ROS are the NOX family [12,13]. All five NOX enzymes are transmembrane oxidoreductases containing dual heme, which span the membrane six times. The electrons from NADPH transfer to the two heme residues via flavin adenine dinucleotide (FAD) and ultimately, to O_2_ to generate ROS [14]. Superoxide anions (O_2_^−^) are generated in this process, which can further react to hydrogen peroxide (H_2_O_2_) or to peroxynitrite (ONOO^−^) in the presence of nitric oxide (NO). Recently, NOX4 gained substantial attention because it is readily distinguished from the other NOX isoforms by its activation, type of ROS released, subcellular localization, tissue-specific expression and influence over signaling pathways.

### 2.1. NOX4

The activation of NOX1–3 depends on phosphorylation and protein-protein interactions of cytosolic subunits, while NOX5 and Duox1 and 2 are Ca^2+^-activated. In contrast, NOX4 is constitutively activated and can produce ROS in the absence of cytosolic subunits, due to the unique intrinsically-activated NOX4 dehydrogenase (DH) domain, which promotes the constitutive electrons transfer from NADPH to FAD [15]. ROS generation by NOX enzymes occurs through electrons transfer from NADPH to O_2_ and thus yields O_2_^−^. While NOX1–3 and NOX5 appear to release O_2_^−^, NOX4 predominantly produces H_2_O_2_. Preferential production of H_2_O_2_ by NOX4 is attributed to a highly conserved histidine in the third extracytosolic loop (E-loop) of NOX4 that accelerates spontaneous dismutation of superoxide to form H_2_O_2_ before it leaves the enzyme [16]. NOX4 directly interacts with p22^phox^ [17], which is a prerequisite for H_2_O_2_ generation [18]. The subcellular localization of NOX4 has been reported in nucleus [19,20], focal adhesions [21], endoplasmic reticulum (ER) [22], plasma membrane (PM) [23] and mitochondria [24], depending on specific cell types [14,25]. Recently, another report shows that NOX4 protein contains a 73 amino acids-long mitochondrial localization signal at the N-terminus [26]. The expression of NOX4 in ECs in vivo is higher than other NOX isoforms [27]. However, the cellular localization and function of NOX4 in EC need further investigations.

Compelling evidence demonstrates that NOX4 and its generated H_2_O_2_ play an important role in cell proliferation, migration, apoptosis and oxygen sensing, which has been reviewed in detail elsewhere [28]. NOX4 regulated specific signaling pathways and cellular function depends on the level of NOX4 expression, the intracellular location and the cell types. Low level H_2_O_2_ derived from NOX4 can activate mitogen-activated protein kinase (MAPK) family members, the transforming growth factor-β1 (TGF-β1)/SMAD2/3 pathway [29] or phosphatidylinositol 3-kinase (PI3K)/ Protein Kinase B (Akt) signaling [30] to promote cell proliferation. In contrast, NOX4 also plays a negative role in hepatocyte proliferation and inhibits liver cancer progression [31]. Increasing evidence suggests that NOX4 participates in promoting cell migration in different cells, which has been reviewed in detail elsewhere [28]. Both cell proliferation and cell migration are crucial during angiogenesis and vascular development.

### 2.2. The Role of NOX4 in Angiogenesis

Angiogenesis is a tightly regulated multistage process, including vessel sprouting, lumen formation and maturation [32]. Upon pro-angiogenic stimulation, ECs firstly sprout from the pre-existing vascellum after the degradation of extracellular matrix (ECM). Afterwards, these ECs undergo proliferation, migration and differentiation and recruit smooth muscle cells (SMCs) or pericytes to cover the newly-formed vessels to promote their maturation. The essential role of NOX4 in angiogenesis has been the subject of research for years. Hepatopulmonary syndrome (HPS) affects 10–30% of patients with cirrhosis and portal hypertension, and pulmonary angiogenesis contributes to the development of HPS [33]. Two NOX4 single nucleotide polymorphisms (SNPs) (rs585197 and rs2164521) have been found in patients with cirrhosis being evaluated for liver transplantation [33]. NOX4^−/−^ mice exhibit attenuated angiogenesis, reduction of endothelial nitric oxide synthase expression, nitric oxide production and heme oxygenase-1 (HO-1) expression [34]. In contrast, endothelial-specific NOX4 transgenic mice exhibit enhanced angiogenesis and blood flow recovery under ischemia in an eNOS-dependent manner [35]. Expression studies suggest that NOX4 expression predominates in ECs [36] and is lower in the smooth muscle layer of the vessel [34]. It is noted that NOX4 expression in SMCs can be increased in response to injury and decreased under normal conditions in the rat carotid artery [37]. Moreover, NOX4 is an important source of ROS in human ECs [35]. Mechanistic studies indicate that NOX4 promotes angiogenic responses, at least partly, via enhanced activation of receptor tyrosine kinases and the downstream extracellular signal-regulated kinase (ERK) pathway. NOX4 expression also promotes proliferation and migration of endothelial cells and reduces serum deprivation-induced apoptosis [38].

### 2.3. NOX4 Signaling Pathways and Regulation in Angiogenesis

NOX4-mediated angiogenesis is regulated by many factors, including hypoxia, ischemia, vascular endothelial growth factor (VEGF), fibroblast growth factor-1 and -2 (FGF-1 and FGF-2), tumor necrosis factor (TNF)-related apoptosis-inducing ligand (TRAIL) and TGF-β1.

#### 2.3.1. Hypoxia

Accumulating lines of evidence suggest that hypoxia promotes angiogenesis by increasing NOX4 expression in different cells. In lung tissue from mice and isolated pulmonary artery smooth muscle cells (PASMCs), hypoxia rapidly enhances NOX4 mRNA and protein levels [39]. Overexpression of hypoxia inducible factor 1 alpha (HIF-1α) increases NOX4 expression, while HIF-1α depletion prevents this response [40]. Induction of NOX4 by HIF-1α contributes to maintain ROS levels after hypoxia and hypoxia-induced proliferation of human pulmonary artery SMCs (HPASMCs). Interestingly, exposure of human pulmonary artery endothelial cells (HPAECs) to hyperoxia also enhances mRNA and protein expression of NOX4, and NOX4 siRNA attenuates hyperoxia-induced ROS production, cell migration and capillary tube formation [20]. In cardiac microvascular endothelial cells (CMECs), NOX4 plays a vital role against hypoxia/reoxygenation (H/R) injury by inhibiting apoptosis and promoting migration and angiogenesis via inhibition of prolyl hydroxylase 2 (PHD2)-dependent upregulation of HIF-1α/VEGF proangiogenic signaling in vitro [41]. Hypoxia also leads to a 30–50% increase in angiogenesis and cell migration by signal transducer and activator of transcription 3 (STAT3) phosphorylation in glioblastoma cells [42]. Activated STAT3 leads to HIF-1α and VEGF expression and angiogenesis. In addition, the expression of NOX4 is increased at both mRNA and protein levels in hypoxic glioblastoma cells [43]. The elevated ROS production by NOX4 plays a vital role for STAT3 activation and angiogenesis in hypoxic glioblastoma cells.

Mounting evidence demonstrates that NOX4 plays an important role in the development of pulmonary vascular remodeling and hypertension caused by hypoxia. NOX4 is exclusively upregulated in the pulmonary arterial vessels of mice with chronic exposure to hypoxia and in the vascular lesions of patients with idiopathic pulmonary arterial hypertension (IPAH) [39]. In isolated PASMCs, the expression of NOX4 is increased after exposure to hypoxia in vitro. In another mouse model, chronic intermittent hypoxia (CIH)-induced pulmonary hypertension is associated with increased lung levels of the NOX4 and p22^phox^, reduced NO bioavailability, increased activity of platelet-derived growth factor receptor β (PDGFRβ) and downstream effector, Akt kinase [44]. On the contrary, activation of peroxisome proliferator–activated receptor γ (PPARγ) with the synthetic ligand rosiglitazone attenuates hypoxia-induced increases in mouse lung NOX4 expression, ROS generation and pulmonary vascular remodeling and hypertension [45]. Hypoxia increases the binding of the nuclear factor-κB (NF-κB) subunit, p65, to the NOX4 promoter in HPASMCs, while PPARγ activation reduces the binding of p65 to the NOX4 promoter [46]. Consistent with this notion, the NOX4 inhibitor GKT137831 attenuates hypoxia-induced H_2_O_2_ release, proliferation and TGF-β1 expression and blunts reductions in PPARγ in vitro and in vivo [47]. Collectively, these findings indicate that functional interference with NOX4 may provide a novel therapeutic approach for the treatment of attenuate hypoxia-induced pulmonary hypertension.

#### 2.3.2. Ischemia

Ischemic diseases are characterized by an impaired supply of nutrients and oxygen resulting from narrowed or blocked arteries [48]. Despite considerable advances in the clinic, vascular occlusion and/or microcirculation impairment put patients at constant risk of ischemia and the consequent detrimental effects on quality-of-life and longevity [49]. Blood flow recovery is significantly attenuated in global NOX4^−/−^ mice, as well as in inducible NOX4^−/−^ mice after femoral artery ligation [34]. In contrast, the endothelial-specific NOX4 overexpression mice accelerate blood flow recovery from hind limb ischemia and increase angiogenesis [35]. Tube formation in cultured NOX4^−/−^ lung endothelial cells (LECs) is attenuated and restored by low concentrations of H_2_O_2_, while polyethylene glycol (PEG)-catalase attenuates tube formation in control LECs [34]. Mechanistically, loss of NOX4 leads to a reduction of endothelial nitric oxide synthase (eNOS) expression, nitric oxide (NO) production and heme oxygenase-1 (HO-1) expression. The expression of HO-1 is controlled by the redox-sensitive transcription factor Nuclear factor (erythroid-derived 2)-like 2 (Nrf-2), which is stabilized in response to oxidative stress [50]. H_2_O_2_, derived from vascular NOX4, prevents Nrf2 degradation [34]. Nrf-2-driven HO-1 expression contributes to the protective effects of NOX4. In conclusion, NOX4 protects vascular function through H_2_O_2_ generation, maintenance of NO production and antioxidant HO-1 expression. Consistent with this notion, endogenous EC-derived H_2_O_2_ plays a crucial role in reparative neovascularization in response to ischemia by activating eNOS in ECs [51]. NOX4 is also upregulated in neurons under ischemic conditions and plays a role in ischemia-induced brain angiogenesis [52].

#### 2.3.3. VEGF

VEGF, a well-known target of HIF1α, is one of the most potent angiogenesis growth factors and stimulates proliferation, migration and tube formation of ECs and angiogenesis in vivo [53]. VEGF binding VEGF receptor 2 (VEGFR2) initiates tyrosine phosphorylation of VEGFR2 and activates downstream signaling, including ERK1/2, Akt and eNOS, which contribute to angiogenic-related responses in EC [54]. NOX4 is shown to mediate VEGF–induced angiogenesis. In HUVECs, VEGF-activated VEGFR2 induces Rac1 to form a complex with NOX4, resulting in a burst of ROS that further promotes angiogenesis [55]. Conversely, NOX4 can induce HIF-1α and HIF-1α-dependent VEGF expression and angiogenesis [42,43]. In cardiac microvascular ECs, NOX4 plays a protective role against H/R injury by inhibiting apoptosis and promoting migration and angiogenesis via a PHD2-dependent upregulation of the HIF-1α/VEGF proangiogenic signaling [41]. Similarly, NOX4 promotes tumor angiogenesis through stabilization of HIF-1α and induction of VEGF expression [56].

#### 2.3.4. TRAIL

Tumor necrosis factor (TNF)-related apoptosis-inducing ligand (TRAIL) is a member of the TNF family of cytokines. In human umbilical vein endothelial cells (HUVECs), recombinant TRAIL induces a proangiogenic phenotype, which includes both early (increase in migration, invasion and proliferation) and late (differentiation into vascular cords) angiogenic events in vitro [57]. More importantly, TRAIL is angiogenic to a degree comparable to VEGF. Using Trail^−/−^ mice, the authors demonstrate that TRAIL plays an important role in ischemia-induced angiogenesis in vivo. The angiogenic effect of TRAIL on human microvascular endothelial cell-1 cells lies downstream of FGF-2. Furthermore, TRAIL also promotes SMCs proliferation after arterial injury [58]. Recently, a report demonstrates that TRAIL promotes angiogenesis in vitro by modulating H_2_O_2_, eNOS phosphorylation at Ser-1177 and NO production via NOX4 [49].

#### 2.3.5. TGF-β1

Transforming growth factor-β1 (TGF-β1) is a multifunctional growth factor, which plays a vital role in many biological processes including embryonic development, cell proliferation, migration, extracellular matrix production and differentiation of a variety of cell types [29]. TGF-β1 induces the expression of NOX4 and ROS-dependent proliferation in human pulmonary artery SMCs (HPASMCs) [59] and human airway SMCs (HAWSMCs) [60]. TGF-β1 promotes NOX4 expression through the activation of SMAD2/3 and ERK1/2 [59]. In another study, TGF-β1 enhances the phosphorylation of SMAD2 and ERK1/2 to promote proliferation of human microvascular endothelial cells (HMECs) [61]. The activator protein (AP)-1/SMAD binding box located between 3.97 kb and 4.76 kb upstream of the transcriptional start site (TSS) of the human NOX4 promoter is fundamental for the transcription of NOX4 gene induced by TGF-β1 in human lung fibroblasts [62]. TGF-β1 also stimulates NOX4 expression and ROS formation in ECs via the SMAD2 pathway to promote angiogenesis, which is dependent on TGF-β1 receptors I activin receptor-like kinase 5 (ALK5) activity [29]. In murine heart ECs (MHECs) from NOX4-deficient mice, TGF-β1-induced cell proliferation, migration and tube formation are abolished. In vivo, TGF-β1-induced angiogenesis is markedly reduced in NOX4 knockout mice.

**Figure 1:**
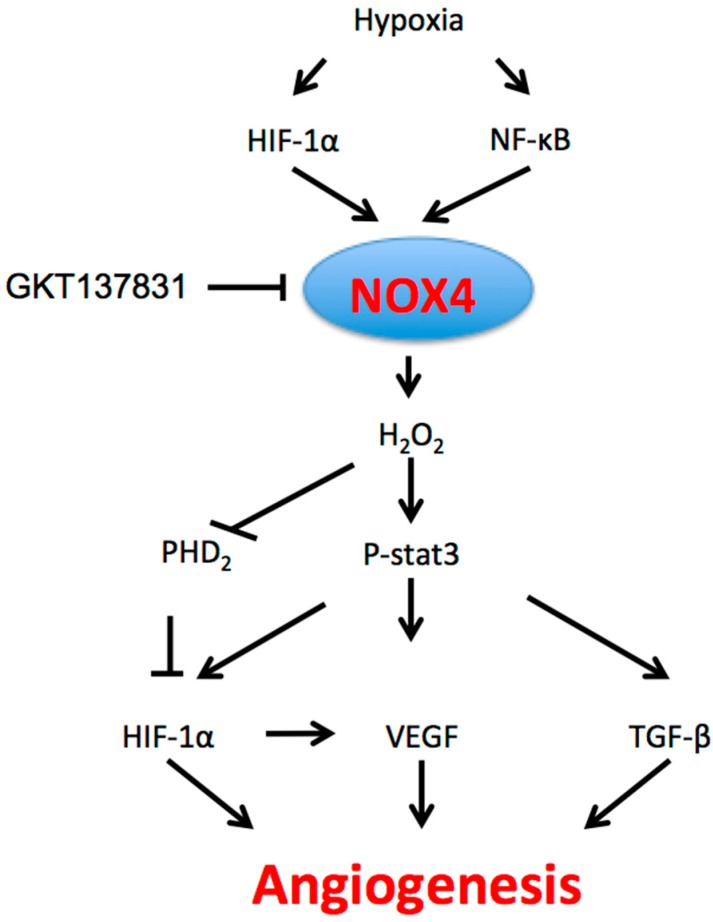
The role of NOX4 in angiogenesis. Hypoxia through HIF-1α and NF-κB induces NOX4 expression; NOX4 generates cytosolic ROS, which in turn activate multiple intracellular angiogenic pathways, including STAT3, VEGF and TGF-β signaling. ROS: reactive oxygen species; STAT3: signal transducer and activator of transcription 3; VEGF: vascular endothelial growth factor; TGF-β: transforming growth factor-β.

On the other hand, NOX4 regulates ischemia-induced angiogenesis through H_2_O_2_^−^ and TGF-β1-mediated cell signaling pathways in endothelial specific NOX4 transgenic mouse lines [63]. Application of TGF-β1 increases both VEGFR2 and eNOS expression levels, which are critical for angiogenesis. Targeting NOX4 with a novel inhibitor GKT137831 [2-(2-chlorophenyl)-4-[3-(dimethylamino)phenyl]-5-methyl-1H-pyrazolo[4,3-c]pyridine-3,6(2H,5H)-dione] attenuates hypoxia-induced H_2_O_2_ release, proliferation and TGF-β1 expression in human pulmonary artery endothelial cells (HPAECs) and pulmonary arterial smooth muscle cells (HPASMCs) in vitro [47]. Therefore, TGF-β1 can serve as both the upstream and downstream of NOX4 signaling (Figure 1). NOX4-derived angiogenesis is also regulated by many other factors: ubiquitination of p300-histone acetyltransferase (p300-HAT) [64], prostacyclin [65], notch [32], insulin [66], exercise [67], chronic stresses [68] and brain-derived neurotrophic factor (BDNF) [69].

### 2.4. Role of NOX4-Mediated Angiogenesis in Cancer

Angiogenesis is necessary for the invasive growth and metastasis of tumors and is an important target in the control of cancer progression [70]. Compelling evidence demonstrates that NOX4 and its generated ROS have a close relation to tumor angiogenesis in different cancers. In a carcinogen 3-methylcholanthrene (MCA)-induced fibrosarcoma mice model, there is a significant 38% reduction in tumor vascularization in fibrosarcomas of NOX4^−/−^ mice [56]. The accumulation of HIF-1α and the expression of the HIF-1α-dependent pro-angiogenic genes such as VEGF-A, glucose transporter 1 (GLUT-1) and adrenomedullin are indeed significantly attenuated in tumors of NOX4^−/−^ mice compared to wild type (WT) mice. HIF1α is a key transcription factor of angiogenesis in solid tumors [71,72] and tumors lacking HIF-1α show significantly reduced vascularization [73]. NOX4 promotes tumor angiogenesis by stabilization of HIF-1α and induction of VEGF expression [56]. In von Hippel Lindau (VHL)-deficient renal cell carcinoma (RCC), NOX4 also promotes renal tumorigenesis in a similar signal pathway via nuclear accumulation of HIF-2α, not HIF-1α [74]. HIF1α expression is biased toward HIF2α [75], whose transcriptional activity is dependent on NOX4 in VHL-deficient RCC [76]. Stable NOX4 knockdown by shRNA significantly reduces ROS production and suppresses glioblastoma cells proliferation and invasion and tumor-associated angiogenesis [77]. Taken together, NOX4 is implicated in tumor angiogenesis of different cancer types, including fibrosarcoma, VHL-deficient RCC, glioblastoma and human astroglioma [78]. Therefore, NOX4 might be a promising target for anti-angiogenic tumor therapy.

## 3. The Thioredoxin System

The thioredoxin (TRX) system belongs to thiol-disulfide oxide reductase that can decrease the level of ROS in cells. The antioxidant function of the TRX system is through reducing peroxiredoxins (Prx) to scavenge ROS [79]. The TRX system can be classified into different isoforms according to the subcellular localization. The TRX1 isoform is distributed in the cytoplasm and nucleus, and the TRX2 isoform is specifically identified in the mitochondria. Homozygous knockout of any isoform in mice can cause early embryonic lethality [80,81]. TRX molecules can be detected throughout the whole process of development, revealing that the TRX system is necessary for life [82,83,84]. TRX proteins contain a conserved active site Trp-Cys-Gly-Pro-Cys [85] that reduces ROS and other oxidized proteins. Using the electron donor NADPH, TRX reductase-1(TRXR1) and TRX reductase-2 (TRXR2) can regenerate TRX1 and TRX2. The catalytic motif of TRX isoforms can be modulated by thioredoxin-interacting protein (TXNIP), inhibiting the function of TRX isoforms to decrease disulfides of the substrates and withstand reversible oxidation. It has been confirmed in several cell lines in vitro [86,87,88], but the results have not been confirmed in vivo [89,90]. All of these traits indicate that TXNIP belong to the endogenous inhibitors of the TRX system.

TRX1 is ubiquitously expressed. TRX2 precursor protein has a mitochondrial localization sequence, and the mature TRX2 protein is localized in mitochondria [91]. In mitochondria, the TRX2 system includes TRX2, TRX2 reductase (TRXR2) and TRX2 dependent PRX3. TRX2 is widely expressed in human tissues, especially enhanced expression in metabolically exuberant organs, like heart, brain and liver [92]. TRX1/2 regulate their target proteins to affect cell growth, apoptosis, inflammatory response and other physiological processes [93]. The redox status of the cysteine groups in certain nucleus transcription factors directly affects their DNA binding capacity, and TRX1 in the nucleus participates in the maintenance of the reduction state of the cysteine residues to enhance the transcription factor activity. Similar to TRX1, TRX2 regulates the transcription factor NF-κB [94] and apoptosis signaling kinase-1 (ASK1). Both TRX1 and TRX2 also regulate ASK1, but in distinct mechanisms. TRX1 interacts with ASK1, promoting ASK1 ubiquitination and degradation to suppress ASK1-mediated apoptosis [95,96]. TRX2 can bind to ASK1 located in the mitochondria and block its activity. Auranofin is a metal phosphine complex initially developed for treatment of rheumatoid arthritis, which has been widely used as an inhibitor of TRX2 to promote ECs apoptosis [97]. TXNIP can also bind to the reduced TRX2, reducing its activity [98]. In the normal growth state of the cells, TXNIP is mainly located in the nucleus, while TRX2 binds ASK1 to block its protein kinase activity in mitochondria [99]. When the cells are under oxidative stress, TXNIP is localized from the nucleus to mitochondria and competes with ASK1 to bind TRX2, causing ASK1 to change from an inhibited state to an active state to induce cell apoptosis. That suggests TRX2 is an endogenous inhibitor of ASK1. Consistently, TRX2 knockdown in EC promotes ASK1 activation and cell apoptosis [99]. Conversely, ECs isolated from TRX2-TG show more resistance to ASK1 activation-induced oxidative stress and apoptosis [100].

### 3.1. TRX1/2 and Angiogenesis

**Figure 2:**
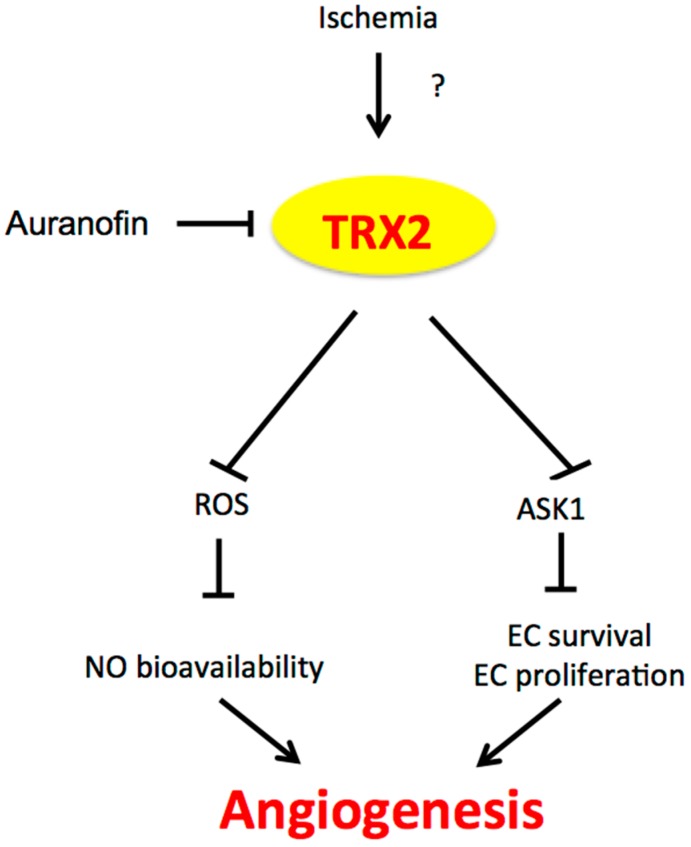
The role of TRX2 in angiogenesis. Ischemia induces TRX2 expression by an unknown mechanism. TRX2 in the mitochondria inhibits mitochondrial ROS production and mitochondrial ASK1-mediated apoptosis, leading to increased NO bioavailability and EC survival/proliferation and augmented angiogenesis. TRX2: Thioredoxin 2; ASK1: apoptosis signaling kinase-1; EC: endothelial cell.

TRX1/2 in ROS scavenging, anti-apoptosis and NF-κB activation implicate their potential function in angiogenesis. In this regard, TRX2 is better understood than TRX1. EC-specific transgenesis of TRX2 (TRX2-TG) has been constructed [101]. By reducing oxidative stress, EC-specific transgenesis of TRX2 (TRX2-TG) reduces atherosclerotic lesions at aortic roots and improves aortic EC function in an ApoE-deficient mouse model [101]. Using TRX2-TG mice, the critical role of TRX2 is to enhance ischemia-mediated arteriogenesis and angiogenesis by increasing NO bioavailability and decreasing apoptosis in EC [100]. The phenotype of TRX2-TG mice is similar to the EC-specific eNOS transgenic mice (eNOS-TG). More importantly, expression of TRX2 in eNOS-KO mice partly rescues the angiogenic defects of the eNOS-KO mice, indicating that that TRX2-augmented NO bioactivity contributes significantly to enhanced angiogenesis in TRX2-TG mice [100]. It is TRX2, but not TRX1, that is downregulated in ischemia. Ischemia appears to regulate TRX2 at the transcriptional level. Therefore, TRX2 plays a critical role in ischemia-mediated arteriogenesis and angiogenesis by decreasing oxidative stress and increasing NO bioactivity (Figure 2).

Apoptosis and cell survival are critical components in angiogenesis and vascular remodeling, which can be regulated by the TRX system. TRX1/2 inhibit apoptosis in both a redox-dependent (by scavenging ROS enhancing NO activity) and a redox activity independent manner (by inhibiting ASK1) [96]. This was elucidated by crossing TRX2-TG with eNOS-deficient or ASK1-deficient mice [100]. Conversely, overexpression of ASK1 in ECs inhibits cell migration and vascular network formation induced by VEGF [102]. Of note, ASK1-interating protein-1(AIP1)-deficient mice have enhanced ischemia and inflammatory angiogenesis in ischemic hind limb and sponge granuloma models [103].

The transcription factor NF-κB regulates various angiogenesis-related genes and proteins, including Akt activation of VEGF, which plays an important role in ECs proliferation, migration and survival [104,105]. NF-κB can directly promote vascular sprouting and neovascularization. TRX1 can be transferred from the cytoplasm to the nucleus, reducing the cysteine residues of NF-κB and activator protein-1(AP-1) nucleoprotein to enhance their transcriptional activity [106,107]. The TRX2 system also regulates NF-κB activity by a yet-to-be-defined mechanism. It is certain that NF-κB contributes to TRX1/2-mediated angiogenesis.

### 3.2. Role of TRX1/2-Mediated Angiogenesis in Cancer

Compelling evidence from both clinical and laboratory studies demonstrates that the TRX system presents as potential target for anticancer drug development [108,109,110]. Enhanced levels of the TRX system are observed in many aggressive tumors [111,112,113]. Moreover, transfection with dominant-negative mutant TRX or depletion of TRXR results in retardation in tumor progression, metastasis and tumor-derived angiogenesis [114,115,116,117].

Recently some cationic triphenylmethanes such as brilliant green (BG) and gentian violet have shown potent antitumor and antiangiogenic activity in mice and humans [118,119]. Mechanistic studies indicate that triphenylmethane dyes induce TRX2 oxidized and degraded by mitochondrial Lon protease [120]. BG can kill cells at nanomolar concentrations and target mitochondrial TRX2, which was oxidized and degraded. In HeLa cells, TRX2 down-regulation by siRNA results in increased sensitivity to BG, whereas for fibroblasts, the same treatments have no effect. BG accumulates in mitochondria and causes a rapid and dramatic decrease in mitochondrial TRX2 protein. Treatment with BG causes oxidation of both TRX1 and TRX2, followed by release of cytochrome c and apoptosis-inducing factor from the mitochondria into the cytosol. Furthermore, this treatment results in an elevation of the mRNA level of Lon protease, a protein quality control enzyme in the mitochondrial matrix, suggesting that the oxidized TRX2 may be degraded by Lon protease. Recently, we have shown that auranofin, a metal phosphine complex, decreases cellular survival protein TRXR2, TRX2 and transcription factor NF-κB whereas increases stress signaling p38MAPK, leading to EC apoptosis at high doses (≥1 μΜ) [97]. In another study, we show that 1,2-bis(methylsulfonyl)-1-[(methylamino)carbonyl] hydrazine (101MDCE), an analog of laromustine that generates only methyl isocyanate, activates ASK1-JNK/p38 signaling in EC [121]. Mechanistically, methyl isocyanate induces dissociation of ASK1 from Trx1 either directly by carbamoylating the critical Cys groups in the ASK1-Trx1 complex or indirectly by inhibiting TRXR1. Our study supports that 101MDCE induces EC death through a non-apoptotic (necroptotic) pathway leading to inhibition of angiogenesis in vitro. Furthermore, one preliminary study finds that TRX2-transgenic mice have a slightly higher incidence of cancer than wild-type mice at old age (24–26 months) [122]. Therefore, the anti-tumor effects of anti-TRX system agents may be largely due to their anti-angiogenic activities. However, the role of TRX1/2-mediated angiogenesis in cancer is not well studied at this moment. The potential effect of TRX1/2-mediated angiogenesis in cancer tumor development in humans needs to be very carefully addressed.

## 4. The Potential Cross-Talk between NOX4 and TRX2 in Angiogenesis

**Figure 3:**
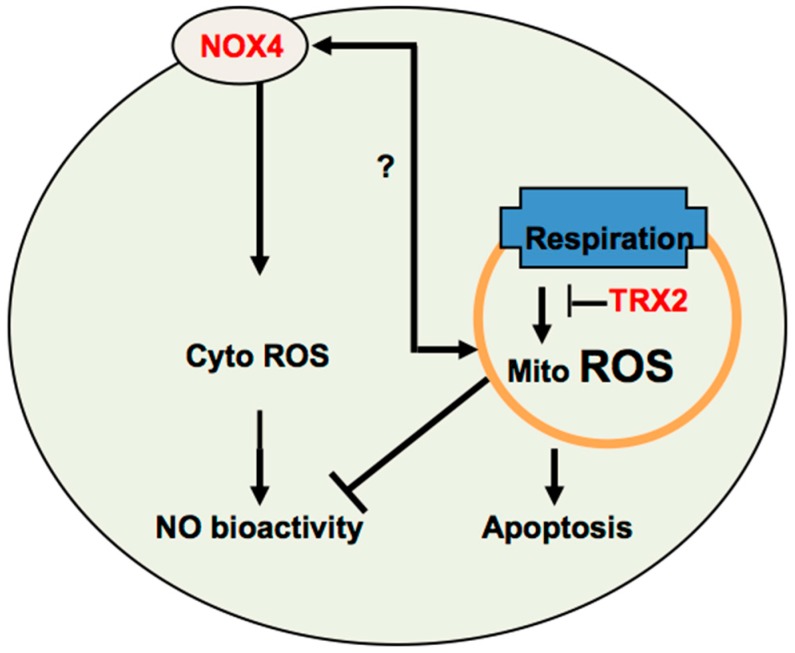
Cross-talk between NOX4 and TRX2-mediated ROS signaling. It is plausible that cytosolic ROS enhance, while mitochondrial ROS inhibit angiogenesis. NOX-generated cytosolic ROS induce angiogenesis, in part, by increasing NO activity. TRX2 prevents mitochondrial ROS-mediated NO dysfunction and EC apoptosis to increase angiogenesis. It is unknown how NOX4 and TRX2 cross-talk. A low level of mitochondrial ROS may upregulate NOX4, which in turn is translocated into mitochondria to induce more ROS production in mitochondria.

The similarities and differences between NOX4 and TRX2-mediated angiogenesis can be readily compared because both NOX4 [35] and TRX2 [100,101] endothelial-specific expression mice have been generated. Both NOX4 and TRX2 play import roles in hindlimb ischemia-induced angiogenesis. TRX2 maintains EC function by two parallel pathways: scavenging ROS to increase NO bioavailability and inhibiting ASK1 activity to enhance EC survival [100]. Similarly, NOX4 promotes angiogenesis and recovery from hypoxia through enhancing eNOS activation and blocking ASK1 activity [35]. However, TRX2 and NOX4 regulate these two common targets (NO and ASK1) by distinct mechanisms. ECs overexpressing TRX2 may regulate NO bioavailability without significant effects on eNOS expression, phosphorylation and/or enzymatic activity. In cultured ECs overexpressing NOX4, eNOS protein expression and activity significantly increase. Endothelial eNOS is a key regulator of angiogenesis [123]. For ASK1, TRX2 directly binds to mitochondria-located ASK1 and blocks mitochondrial apoptotic signaling. NOX4-generated ROS also stimulate phosphorylation of Akt, which in turn inactivates ASK1 by phosphorylating ASK1 on Ser-83, leading to an antiapoptotic effect [124]. Therefore, it is plausible that the TRX2 and NOX4 pathways synergistically promote angiogenesis. However, TRX2 and NOX4 genes are distinctly regulated under pathogenesis. Expression of TRX2 is drastically reduced in response to hypoxia in vitro and in vivo [125]. In contrast, NOX4 increases in response to hypoxia in vitro and in vivo [35]. Similarly, hypoxia inhibits TRX2 expression, but promotes NOX4 expression of pulmonary hypertension [39,125]. The reciprocal expression of TRX2 and NOX4 under pathological conditions suggests that TRX2 and NOX4 exhibit synergistic effects in physiological angiogenesis when both TRX2 and NOX4 are expressed. However, NOX4 may play a more predominant role for pathological angiogenesis with the reduced or absent of TRX2. Moreover, NOX4 may compensate TRX2 function under pathological settings. In the heart tissues from cardiac-specific TRX2 knockout mice, gene analyses by NanoString technology show that NOX4 expression increases [126]. Consistent with our findings, expression of a dominant negative C93S TRX2 mutant that mimics TRX2 oxidation exacerbated hypoxia-induced increases in HPASMC H_2_O_2_ levels and cell proliferation [125], which may be a result of damaged TRX2-derived increase of NOX4 expression. It is unclear how TRX2 deficiency induces NOX4 expression, and it is likely that mitochondria-derived ROS could directly activate NOX4 or indirectly increase NOX4 expression. It has also been reported that NOX4 can be located in the mitochondria where NOX4 may regulate mitochondria ROS generation. NOX4 and TRX1/2 are also regulated by similar angiogenic factors such as hypoxia, ischemia and VEGF. NOX4-mediated angiogenesis is also regulated by many other factors, including FGF-1, FGF-2, TRAIL and TGF-β1. An important question is to address if these angiogenic factors contribute to TRX2-mediated angiogenesis. The mechanism responsible for the cross-talk between NOX4 and TRX2 requires further investigations (Figure 3).

## 5. Conclusions

The effects of ROS on angiogenesis are dependent on the amount, species and site of production, as well as the balance of pro-oxidant and antioxidant enzyme activity. The mechanisms of NOX4 in all forms of angiogenesis have been the subject of research for years. However, NOX4-specific inhibitors or activators are urgently required. Although the role of TRX2 in ischemia-induced angiogenesis has been studied, further research is required to define the mechanism of TRX2 regulation in various pathological settings. In spite of increasing lines of evidence suggesting that NOX4 and TRX2 may have a cross-talk in angiogenesis, more studies are needed to illustrate the underlying mechanisms, which may provide that NOX4 and TRX2 are potential therapeutic targets for the treatment of angiogenesis-dependent diseases.
